# Supplementary material for: Meiotic cellular rejuvenation is coupled to nuclear remodeling in budding yeast
Source: eLife. 2019 Aug 9;8:e47156. doi: 10.7554/eLife.47156 (PMC6711709; doi:10.7554/eLife.47156)
Supplement: Figure 1—figure supplement 1—source data 1. [file elife-47156-fig1-figsupp1-data1.pdf]

	Percent of cells
Aggregate inside nucleus	96
Aggregate outside nucleus	4
