# Supplementary material for: Meiotic cellular rejuvenation is coupled to nuclear remodeling in budding yeast
Source: eLife. 2019 Aug 9;8:e47156. doi: 10.7554/eLife.47156 (PMC6711709; doi:10.7554/eLife.47156)
Supplement: Figure 2—source data 1. [file elife-47156-fig2-data1.pdf]

Time of nucleolar sequestration relative to anaphase II onset (min)	Percent of aged cells
-45	0
-30	0
-15	0
0	2
15	62
30	98
45	100
60	100
