# Supplementary material for: Meiotic cellular rejuvenation is coupled to nuclear remodeling in budding yeast
Source: eLife. 2019 Aug 9;8:e47156. doi: 10.7554/eLife.47156 (PMC6711709; doi:10.7554/eLife.47156)
Supplement: Figure 2—source data 2. [file elife-47156-fig2-data2.pdf]

	Number of generations (age)		
	0-4	5-8	9+
percent Nsr1 sequestered	21.63	32.06	56.10
	21.96	27.39	63.11
	24.93	29.87	26.56
	29.21	20.75	56.35
	28.98	22.11	64.57
	17.70	53.80	20.92
	15.54	34.63	26.05
	18.03	43.71	47.01
	11.55	18.97	58.99
	28.42	41.56	39.36
	8.47	26.26	47.33
	24.76	47.48	84.58
	14.79	34.44	17.06
	17.78	46.08	40.87
	24.57	55.88	25.95
	19.58	36.50	59.69
	31.83	24.23	55.04
	20.63	28.12	29.14
	26.16	26.12	28.09
	15.22	47.19	27.82
	57.45	61.08	22.54
	26.32	22.70	51.33
	22.78	28.83	21.03
	30.45	59.56	23.56
	19.26	60.35	27.72
	23.22	61.22	51.27
	21.09	26.51	23.60
	14.38	37.41	32.75
	26.35	67.65	67.82
	20.39	66.01	46.38
	19.87	44.18	14.87
	13.57	33.20	32.20
	33.24	20.62	14.70
	21.77	11.85	28.40
	34.42	21.26	24.13
	9.08	21.35	49.65
	24.44	23.39	73.66
	21.63	34.47	45.59
	22.33	26.60	78.81
	13.71	30.46	43.17
	21.74	34.08	32.02
	14.57	30.15	21.17
	39.00	10.62	28.08
	6.65	25.44	53.95
	33.30	42.15	31.36
	22.39	36.81	42.61
	39.53	58.05	67.97
	25.74	28.22	53.36
	18.20	34.21	76.65
	15.28	40.19	
		34.49	
		65.74	
		32.98	
