# Supplementary material for: Meiotic cellular rejuvenation is coupled to nuclear remodeling in budding yeast
Source: eLife. 2019 Aug 9;8:e47156. doi: 10.7554/eLife.47156 (PMC6711709; doi:10.7554/eLife.47156)
Supplement: Figure 2—source data 3. [file elife-47156-fig2-data3.pdf]

	Percent of cells
Nsr1 and Hsp104 foci adjacent	86
Nsr1 and Hsp104 foci not adjacent	14
