# Supplementary material for: Meiotic cellular rejuvenation is coupled to nuclear remodeling in budding yeast
Source: eLife. 2019 Aug 9;8:e47156. doi: 10.7554/eLife.47156 (PMC6711709; doi:10.7554/eLife.47156)
Supplement: Figure 7—source data 1. [file elife-47156-fig7-data1.pdf]

Time of Nup170 clearance relative to vacuolar lysis (min)	Percent of young cells
-120	0.00
-105	0.00
-90	0.00
-75	0.00
-60	0.00
-45	0.71
-30	5.67
-15	17.73
0	85.11
15	97.87
30	99.29
45	99.29
60	99.29
75	99.29
90	99.29
105	99.29
120	100.00
