# Supplementary material for: Meiotic cellular rejuvenation is coupled to nuclear remodeling in budding yeast
Source: eLife. 2019 Aug 9;8:e47156. doi: 10.7554/eLife.47156 (PMC6711709; doi:10.7554/eLife.47156)
Supplement: Figure 7—source data 2. [file elife-47156-fig7-data2.pdf]

Time of protein aggregate clearance relative to vacuolar lysis (min)	Percent of aged cells
-240	0
-225	1
-150	2
-135	3
-120	5
-90	7
-75	9
-60	12
-45	20
-30	31
-15	48
0	99
15	99
30	99
45	99
60	99
75	100
240	100
