# Supplementary material for: Meiotic cellular rejuvenation is coupled to nuclear remodeling in budding yeast
Source: eLife. 2019 Aug 9;8:e47156. doi: 10.7554/eLife.47156 (PMC6711709; doi:10.7554/eLife.47156)
Supplement: Figure 8—source data 1. [file elife-47156-fig8-data1.pdf]

	Number of nuclei with NPC enrichment (% of cells)				
	0	1	2	3	4
WT	74.07	20.37	4.63	0.93	0.00
spo21Δ	0.00	0.00	0.85	4.24	94.92
