# Supplementary material for: Meiotic cellular rejuvenation is coupled to nuclear remodeling in budding yeast
Source: eLife. 2019 Aug 9;8:e47156. doi: 10.7554/eLife.47156 (PMC6711709; doi:10.7554/eLife.47156)
Supplement: Figure 8—source data 2. [file elife-47156-fig8-data2.pdf]

		Number of nuclei with NPC enrichment (% of cells)				
		0	1	2	3	4
Number of PMs	4	97.92	2.08	0.00	0.00	0.00
	3	1.25	96.25	2.50	0.00	0.00
	2	0.00	4.35	93.48	2.17	0.00
