# Supplementary material for: Meiotic cellular rejuvenation is coupled to nuclear remodeling in budding yeast
Source: eLife. 2019 Aug 9;8:e47156. doi: 10.7554/eLife.47156 (PMC6711709; doi:10.7554/eLife.47156)
Supplement: Figure 9—source data 1. [file elife-47156-fig9-data1.pdf]

	Percent of aged cells	
	Aggregate sequestered	Aggregate retained
WT	100	0
spo21Δ	52	48
