# Supplementary material for: Meiotic cellular rejuvenation is coupled to nuclear remodeling in budding yeast
Source: eLife. 2019 Aug 9;8:e47156. doi: 10.7554/eLife.47156 (PMC6711709; doi:10.7554/eLife.47156)
Supplement: Figure 9—source data 2. [file elife-47156-fig9-data2.pdf]

	Percent of young cells	
	Nsr1 sequestered	Nsr1 retained
WT	100	0
spo21Δ	61	39
