# Supplementary material for: Meiotic cellular rejuvenation is coupled to nuclear remodeling in budding yeast
Source: eLife. 2019 Aug 9;8:e47156. doi: 10.7554/eLife.47156 (PMC6711709; doi:10.7554/eLife.47156)
Supplement: Supplementary file 1. [file elife-47156-supp1.docx]

**Table S1. Strain table.**

| **Strain** | **Genotype** |
| --- | --- |
| SK1  wild-type | *ho::LYS2 lys2 ura3 leu2::hisG his3::hisG trp1::hisG* |
| UB3810 | *MAT***a** */MATalpha* *Htb1-mCherry::HISMX6/Htb1-mCherry::HISMX6*  *Nup53-eGFP:KanMX6/Nup53-eGFP:KanMX6* |
| UB9724 | *MAT***a** */MATalpha* *Htb1-mCherry::HISMX6/Htb1-mCherry::HISMX6 Hsp104-eGFP::KanMX6/Hsp104-eGFP::KanMX6 flo8::KanMX6/ flo8::KanMX6* |
| UB11513 | *MAT***a** */MATalpha* *Htb1-mCherry::HISMX6/Htb1-mCherry::HISMX6 Nup170-GFP::KanMX6/Nup170-GFP::KanMX6* |
| UB11821 | *MAT***a** */MATalpha Hsp104-mCherry::NatMX6/Hsp104-mCherry::NatMX6 leu2::pATG8-link-yeGFP-SPO20(51-91)::LEU2/leu2::pATG8-link-yeGFP-SPO20(51-91)::LEU2 flo8::KanMX6/flo8::KanMX6* |
| UB12163 | *MAT***a** */MATalpha flo8::KanMX6/flo8::KanMX6 Hsp104-mCherry::NatMX6/Hsp104-mCherry::NatMX6 his3::VPH1-eGFP::HIS3/ his3::VPH1-eGFP::HIS3* |
| UB12342 | *MAT***a** */MATalpha leu2::pATG8-link-yeGFP-SPO20(51-91)::LEU2/ leu2::pATG8-link-yeGFP-SPO20(51-91)::LEU2 Nup49-mCherry::KanMX6/ Nup49-mCherry::KanMX6* |
| UB12414 | *MAT***a** */MATalpha Ady3::HygB/Ady3::HygB Htb1-mCherry::HISMX6/ Htb1-mCherry::HISMX6 Nup170-GFP::KanMX6/Nup170-GFP::KanMX6* |
| UB12434 | *MAT***a** */MATalpha leu2::pATG8-link-yeGFP-SPO20(51-91)::LEU2/ leu2::pATG8-link-yeGFP-SPO20(51-91)::LEU2 Htb1-mCherry::HISMX6/Htb1-mCherry::HISMX6* |
| UB12436 | *MAT***a** */MATalpha Don1-GFP::KanMX6/Don1-GFP::KanMX6 Nup49-mCherry::KanMX6/Nup49-mCherry::KanMX6* |
| UB12438 | *MAT***a** */MATalpha Don1-GFP::KanMX6/Don1-GFP::KanMX6 Htb1-mCherry::HISMX6/Htb1-mCherry::HISMX6* |
| UB12461 | *MAT***a** */MATalpha Don1::HygB/Don1::HygB* *Nup170-GFP::KanMX6/Nup170-GFP::KanMX6* *Htb1-mCherry::HISMX6/Htb1-mCherry::HISMX6* |
| UB12463 | *MAT***a** */MATalpha Irc10::HygB/Irc10::HygB Nup170-GFP::KanMX6/Nup170-GFP::KanMX6* *Htb1-mCherry::HISMX6/Htb1-mCherry::HISMX6* |
| UB12465 | *MAT***a** */MATalpha ady3::HygB/ady3::HygB irc10::HygB/irc10::HygB* *Nup170-GFP::KanMX6/Nup170-GFP::KanMX6* *Htb1-mCherry::HISMX6/ Htb1-mCherry::HISMX6* |
| UB12932 | *MAT***a** */MATalpha leu2::pARO10-eGFP-h2NLS-L-TM::LEU2/leu2:: pARO10-eGFP-h2NLS-L-TM::LEU2 Nup49-mCherry::KanMX6/ Nup49-mCherry::KanMX6* |
| UB12975 | *MAT***a** */MATalpha Nup170-GFP::KanMX6/ Nup170-GFP::KanMX6 Hsp104-mCherry::NatMX6/Hsp104-mCherry::NatMX6 flo8::KanMX6/flo8::KanMX6* |
| UB13299 | *MAT***a** */MATalpha Nsr1-GFP::KanMX6/ Nsr1-GFP::KanMX6 Hsp104-mCherry::NatMX6/Hsp104-mCherry::NatMX6 flo8::KanMX6/flo8::KanMX6* |
| UB13373 | *MAT***a** */MATalpha ssp1::KanMX6/ ssp1::KanMX6 Nup170-GFP::KanMX6/Nup170-GFP::KanMX6* *Htb1-mCherry::HISMX6/Htb1-mCherry::HISMX6* |
| UB13377 | *MAT***a** */MATalpha spo21::HygB/spo21::HygB Nup170-GFP::KanMX6/Nup170-GFP::KanMX6* *Htb1-mCherry::HISMX6/Htb1-mCherry::HISMX6* |
| UB13473 | *MAT***a** */MATalpha ssp1::KanMX6/ ssp1::KanMX6 leu2::pATG8-link-yeGFP-SPO20(51-91)::LEU2/ leu2::pATG8-link-yeGFP-SPO20(51-91)::LEU2 Nup49-mCherry::KanMX6/ Nup49-mCherry::KanMX6* |
| UB13475 | *MAT***a** */MATalpha ssp1::KanMX6/ ssp1::KanMX6 leu2::pATG8-link-yeGFP-SPO20(51-91)::LEU2/ leu2::pATG8-link-yeGFP-SPO20(51-91)::LEU2 Htb1-mCherry::HISMX6/Htb1-mCherry::HISMX6* |
| UB13497 | *MAT***a** */MATalpha Nup84-GFP::KanMX6/ Nup84-GFP::KanMX6 Htb1-mCherry::HISMX6/Htb1-mCherry::HISMX6* |
| UB13499 | *MAT***a** */MATalpha Nup120-GFP::KanMX6/Nup120-GFP::KanMX6 Htb1-mCherry::HISMX6/Htb1-mCherry::HISMX6* |
| UB13503 | *MAT***a** */MATalpha Pom34-GFP::KanMX6/Pom34-GFP::KanMX6* *Htb1-mCherry::HISMX6/Htb1-mCherry::HISMX6* |
| UB13505 | *MAT***a** */MATalpha Nup188-GFP::KanMX6/Nup188-GFP::KanMX6 Htb1-mCherry::HISMX6/Htb1-mCherry::HISMX6* |
| UB13509 | *MAT***a** */MATalpha Nup49-GFP::KanMX6/Nup49::KanMX6 Htb1-mCherry::HISMX6/Htb1-mCherry::HISMX6* |
| UB13568 | *MAT***a** */MATalpha spo21::HygB/spo21::HygB Hsp104-mCherry::NatMX6/ Hsp104-mCherry::NatMX6 Nup170-GFP::KanMX6/Nup170-GFP::KanMX6 flo8::KanMX6/flo8::KanMX6* |
| UB13583 | *MAT***a** */MATalpha ady3::HygB/ady3::HygB Irc10::HygB/Irc10::HygB leu2::pATG8-link-yeGFP-SPO20(51-91)::LEU2/ leu2::pATG8-link-yeGFP-SPO20(51-91)::LEU2 Nup49-mCherry::KanMX6/ Nup49-mCherry::KanMX6* |
| UB13585 | *MAT***a** */MATalpha ady3::HygB/ady3::HygB Irc10::HygB/Irc10::HygB leu2::pATG8-link-yeGFP-SPO20(51-91)::LEU2/ leu2::pATG8-link-yeGFP-SPO20(51-91)::LEU2 Htb1-mCherry::HISMX6/Htb1-mCherry::HISMX6* |
| UB14391 | *MAT***a** */MATalpha Heh1-3xeGFP::KanMX6/Heh1-3xeGFP::KanMX6 Nup49-mCherry::KanMX6/ Nup49-mCherry::KanMX6* |
| UB14393 | *MAT***a** */MATalpha Heh1-3xeGFP::KanMX6/Heh1-3xeGFP::KanMX6 Htb1-mCherry::HISMX6/ Htb1-mCherry::HISMX6* |
| UB14418 | *MAT***a** */MATalpha Htb1-mCherry::HISMX6/Htb1-mCherry::HISMX6 Hsp104-eGFP::KanMX6/Hsp104-eGFP::KanMX6 flo8::KanMX6/ flo8::KanMX6 spo21::HygB/spo21::HygB* |
| UB14419 | *MAT***a** */MATalpha Htb1-mCherry::HISMX6/Htb1-mCherry::HISMX6 Nsr1-GFP::KanMX6/ Nsr1-GFP::KanMX6 spo21::HygB/spo21::HygB* |
| UB14425 | *MAT***a** */MATalpha Nsr1-GFP::KanMX6/ Nsr1-GFP::KanMX6 Nup49-mCherry::KanMX6/ Nup49-mCherry::KanMX6 spo21::HygB/spo21::HygB* |
| UB14646 | *MAT***a** */MATalpha Nup60-GFP::KanMX6/ Nup60-GFP::KanMX6 Htb1-mCherry::HISMX6/Htb1-mCherry::HISMX6* |
| UB14650 | *MAT***a** */MATalpha Nup159-GFP::KanMX6/ Nup159-GFP::KanMX6 Htb1-mCherry::HISMX6/Htb1-mCherry::HISMX6* |
| UB14652 | *MAT***a** */MATalpha Nup82-GFP::KanMX6/ Nup82-GFP::KanMX6 Htb1-mCherry::HISMX6/Htb1-mCherry::HISMX6* |
| UB14654 | *MAT***a** */MATalpha Nup57-GFP::KanMX6/Nup57-GFP::KanMX6 Htb1-mCherry::HISMX6/Htb1-mCherry::HISMX6* |
| UB15118 | *MAT***a** */MATalpha Nsr1-GFP::KanMX6/ Nsr1-GFP::KanMX6 Htb1-mCherry::HISMX6/Htb1-mCherry::HISMX6* |
| UB15301 | *MAT***a** */MATalpha Ndc1-GFP::KanMX6/ Ndc1-GFP::KanMX6 Htb1-mCherry::HISMX6/Htb1-mCherry::HISMX6* |
| UB15303 | *MAT***a** */MATalpha Nup1-GFP::KanMX6/ Nup1-GFP::KanMX6 Htb1-mCherry::HISMX6/Htb1-mCherry::HISMX6* |
| UB15305 | *MAT***a** */MATalpha Nup2-GFP::KanMX6/ Nup2-GFP::KanMX6 Htb1-mCherry::HISMX6/Htb1-mCherry::HISMX6* |
| UB15307 | *MAT***a** */MATalpha spr3::HygB/ spr3::HygB Nup170-GFP::KanMX6/ Nup170-GFP::KanMX6* *Htb1-mCherry::HISMX6/ Htb1-mCherry::HISMX6* |
| UB15426 | *MAT***a** */MATalpha spr28::HygB/ spr28::HygB Nup170-GFP::KanMX6/ Nup170-GFP::KanMX6* *Htb1-mCherry::HISMX6/ Htb1-mCherry::HISMX6* |
| UB15428 | *MAT***a** */MATalpha spr3::HygB/ spr3::HygB spr28::HygB/ spr28::HygB Nup170-GFP::KanMX6/ Nup170-GFP::KanMX6* *Htb1-mCherry::HISMX6/ Htb1-mCherry::HISMX6* |
| UB15672 | *MAT***a** */MATalpha Nup2-GFP::KanMX6/ Nup2-GFP::KanMX6 Nup49-mCherry::KanMX6/ Nup49-mCherry::KanMX6* |
| UB15890 | *MAT***a** */MATalpha Nup170-GFP::KanMX6/Nup170-GFP::KanMX6 Vph1-mCherry::HisMX6/Vph1-mCherry::HisMX6* |
| UB16708 | *MAT***a** */MATalpha Nsr1-GFP::KanMX6/ Nsr1-GFP::KanMX6 Nup49-mCherry::KanMX6/ Nup49-mCherry::KanMX6 flo8/flo8* |
| UB16710 | *MAT***a** */MATalpha Nsr1-GFP::KanMX6/ Nsr1-GFP::KanMX6 leu2::pATG8-link-mKate-SPO20(51-91)::LEU2/leu2::pATG8-link-mKate-SPO20(51-91)::LEU2 flo8/flo8* |
| UB16712 | *MAT***a** */MATalpha Nsr1-GFP::KanMX6/ Nsr1-GFP::KanMX6 Htb1-mCherry::HISMX6/ Htb1-mCherry::HISMX6 flo8/flo8* |
| UB17338 | *MAT***a** */MATalpha Hta1-mApple::HIS5/ Hta1-mApple::HIS5 rDNA-5xtetO/rDNA pREC8-TetR-GFP::LEU2/ pREC8-TetR-GFP::LEU2 flo8/flo8 * Derived from HY2545* |
| UB17509 | *MAT***a** */MATalpha Hta1-mApple::HIS5/ Hta1-mApple::HIS5 pREC8-TetR-GFP::LEU2/ pREC8-TetR-GFP::LEU2 flo8/flo8 * Derived from HY2545* |
| UB17532 | *MAT***a** */MATalpha Nup49-mCherry::KanMX6/ Nup49-mCherry::KanMX6 rDNA-5xtetO/+ pREC8-TetR-GFP::LEU2/ pREC8-TetR-GFP::LEU2 flo8/flo8 * Derived from HY2545* |
| UB19927 | *MAT***a** */MATalpha* *Htb1-mCherry::HISMX6/Htb1-mCherry::HISMX6 Nup170-GFP::KanMX6/Nup170-GFP::KanMX6 ndt80::LEU2/ndt80::LEU2* |
| UB19229 | *MAT***a** */MATalpha Nup84-GFP::KanMX6/ Nup84-GFP::KanMX6 Htb1-mCherry::HISMX6/Htb1-mCherry::HISMX6 ndt80::LEU2/ndt80::LEU2* |
| UB19752 | *MAT***a** */MATalpha Hsp104-mCherry::NatMX6/Hsp104-mCherry::NatMX6 leu2::pATG8-link-yeGFP-SPO20(51-91)::LEU2/leu2::pATG8-link-yeGFP-SPO20(51-91)::LEU2 flo8::KanMX6/flo8::KanMX6 spr3::HygB/ spr3::HygB* |
| UB19754 | *MAT***a** */MATalpha Hsp104-mCherry::NatMX6/Hsp104-mCherry::NatMX6 leu2::pATG8-link-yeGFP-SPO20(51-91)::LEU2/leu2::pATG8-link-yeGFP-SPO20(51-91)::LEU2 flo8::KanMX6/flo8::KanMX6 spr28::HygB/ spr28::HygB* |
| UB19756 | *MAT***a** */MATalpha Hsp104-mCherry::NatMX6/Hsp104-mCherry::NatMX6 leu2::pATG8-link-yeGFP-SPO20(51-91)::LEU2/leu2::pATG8-link-yeGFP-SPO20(51-91)::LEU2 flo8::KanMX6/flo8::KanMX6 don1::HygB/ don1::HygB* |
| UB19758 | *MAT***a** */MATalpha Hsp104-mCherry::NatMX6/Hsp104-mCherry::NatMX6 leu2::pATG8-link-yeGFP-SPO20(51-91)::LEU2/leu2::pATG8-link-yeGFP-SPO20(51-91)::LEU2 flo8::KanMX6/flo8::KanMX6 ady3::HygB/ ady3::HygB* |
| UB19760 | *MAT***a** */MATalpha Hsp104-mCherry::NatMX6/Hsp104-mCherry::NatMX6 leu2::pATG8-link-yeGFP-SPO20(51-91)::LEU2/leu2::pATG8-link-yeGFP-SPO20(51-91)::LEU2 flo8::KanMX6/flo8::KanMX6 spr3::HygB/ spr3::HygB spr28::HygB/ spr28::HygB* |
| UB19762 | *MAT***a** */MATalpha Hsp104-mCherry::NatMX6/Hsp104-mCherry::NatMX6 leu2::pATG8-link-yeGFP-SPO20(51-91)::LEU2/leu2::pATG8-link-yeGFP-SPO20(51-91)::LEU2 flo8::KanMX6/flo8::KanMX6 irc10::HygB/ irc10::HygB* |
