# Supplementary material for: Meiotic cellular rejuvenation is coupled to nuclear remodeling in budding yeast
Source: eLife. 2019 Aug 9;8:e47156. doi: 10.7554/eLife.47156 (PMC6711709; doi:10.7554/eLife.47156)
Supplement: Supplementary file 2. [file elife-47156-supp2.docx]

**Table S2. Primers used for strain construction.**

| **Construct name** | **Forward primer** | **Reverse primer** |
| --- | --- | --- |
| *HEH1-3xeGFP* | GGAACTCAATGAACCTAAGGATTCCGCTGAAAACAAAATA**cggatccccgggttaattaa** | TTTGAGAAGAGAAAACTACGTTTGAGTTTCATTTTGTGGG**GAATTCGAGCTCGTTTAAAC** |
| *NUP53-eGFP* | AAATAGATTGAATAATTGGTTATTTGGATGGAATGATTTG**ggtgacggtgctggttta** | AATCGCACCAAAGCACTACATTTGGGGGTAAGGTTTTTCA**tcgatgaattcgagc** |
| *NUP84-GFP* | GTATCTGGATCTCGTTGCTCGCACAGCAACCCTTTCGAAT**CGGATCCCCGGGTTAATTAA** | TTACTTAAAATATAAACTTATTCTGCAATACATTAATTGA**GAATTCGAGCTCGTTTAAAC** |
| *NUP120-GFP* | GGTTACTTTAACTGATTTAAGAGATGAGTTACGAGGTCTA**CGGATCCCCGGGTTAATTAA** | ATTTTTTAAATGAAGTATTAATTTACAGTTTATATATTCA**GAATTCGAGCTCGTTTAAAC** |
| *NUP170-GFP* | GAACAGCGGCAATAATTTGGGGATTTGTTTCTACAAAGAA**CGGATCCCCGGGTTAATTAA** | ACGTACATTACCCTGCTATCTATATGTCGAACATGAATTT**GAATTCGAGCTCGTTTAAAC** |
| *POM34-GFP* | TGCATATATGATGAACTCACAGTCCCCAAGGGGTAAAATA**CGGATCCCCGGGTTAATTAA** | TATATAGCTATGGAAAGTATTAAATGTTTTTTTGCTGTTT**GAATTCGAGCTCGTTTAAAC** |
| *NUP188-GFP* | AGACATTAAAGCATTACAAGATTCACTATTCAAGGACGTT**CGGATCCCCGGGTTAATTAA** | ATTATTATATTATGTAGCTTTACATAACTTACAAAATAAG**GAATTCGAGCTCGTTTAAAC** |
| *NDC1-GFP* | GTTTCTAGAAGTGTACGCCTCAGGCAACCCTAATGCTACG**CGGATCCCCGGGTTAATTAA** | ACATGAAATGGGAGGAGGGGTGCTCCTCGGTTGAATTGTA**GAATTCGAGCTCGTTTAAAC** |
| *HSP104-mCherry* | CGATAATGAGGACAGTATGGAAATTGATGATGACCTAGAT**CGGATCCCCGGGTTAATTAA** | ATTCTTGTTCGAAAGTTTTTAAAAATCACACTATATTAAA**GAATTCGAGCTCGTTTAAAC** |
| *ssp1Δ* | GGCGACACAAAATCATGAAG | TGATGTTTATGTATAGATCTCTCGA |
| *NSR1-GFP* | AAATACCGCTTCTTTCGCTGGTTCAAAGAAAACATTTGAT**CGGATCCCCGGGTTAATTAA** | AAGAGAAAAAATTGAAATTGAAATTCATTTCATTTTCTCA**GAATTCGAGCTCGTTTAAAC** |
| *don1Δ* | TTTGGCTGGTATTTAAACACAAGTAAGAGAAGCATCAAAC**CGGATCCCCGGGTTAATTAA** | GCACTTTGCCGAAAGAGTTAATAAACATTACCGCTATACA**GAATTCGAGCTCGTTTAAAC** |
| *DON1-GFP* | AAAGCAGGTTCATCCATCTAGACAAGAATTAAGTTTTACG**CGGATCCCCGGGTTAATTAA** | GCACTTTGCCGAAAGAGTTAATAAACATTACCGCTATACA**GAATTCGAGCTCGTTTAAAC** |
| *ady3Δ* | TTTTGAATGGGATAGTTGAATACAACAAACTTCTCCGAAT**CGGATCCCCGGGTTAATTAA** | ACACCATTGAATATATTAGTTCTAAATAAAAAAAAAAAAG**GAATTCGAGCTCGTTTAAAC** |
| *irc10Δ* | AGTCTGCGGTATAATCACCTGGCCTAGTGCTTTTTCAATC**CGGATCCCCGGGTTAATTAA** | CTATATGTCAAGGGTGTCCCAAAATAAAAACTAACAGTAC**GAATTCGAGCTCGTTTAAAC** |
| *NUP60-GFP* | TGAAAATAAAGTTGAGGCTTTCAAGTCCCTATATACCTTT**CGGATCCCCGGGTTAATTAA** | GGGCTATACGGTAATTATGTCACGGCTAAAATTTTCATTA**GAATTCGAGCTCGTTTAAAC** |
| *NUP159-GFP* | GCAAATTGGTGATTTCTTCAAAAATTTGAACATGGCAAAA**CGGATCCCCGGGTTAATTAA** | TTATTAACGGCACTAACAACGTACATATAGCTAAATATCA**GAATTCGAGCTCGTTTAAAC** |
| *NUP82-GFP* | ATTGTTACAAGTTTCTCAGGAATTTACTACTAAAACTCAA**CGGATCCCCGGGTTAATTAA** | TAGCGTACATATATGATAGCAGACTATGCAAGTCGCTTAC**GAATTCGAGCTCGTTTAAAC** |
| *NUP57-GFP* | GAAAGATGCTGCAATTGTAAAAAAATATAAAAATAAAACG**CGGATCCCCGGGTTAATTAA** | CGATCTTTATACAATTCAGTCATTGATTTAAGTAACCTGA**GAATTCGAGCTCGTTTAAAC** |
| *NUP1-GFP* | GGCGAACAGAAAGATTGCAAGAATGAGGCACTCTAAAAGG**CGGATCCCCGGGTTAATTAA** | TTCAGAAAAGCAACACAATACCTAATTACATAACCGATAT**GAATTCGAGCTCGTTTAAAC** |
| *NUP2-GFP* | ATTTACGAAAGCTATTGAAGATGCTAAAAAAGAAATGAAA**CGGATCCCCGGGTTAATTAA** | AGGGTTCTATTCTATTTAAAATTGTTAACTGTATTTACTC**GAATTCGAGCTCGTTTAAAC** |
| *spr3Δ* | TAAAAACCTAAAATTCCTTTTGCGTCATTGAATTTTTATT**CGGATCCCCGGGTTAATTAA** | TTGCGCGAAATTATTGGCTTTTTTTTTTTTTTAATTAATA**GAATTCGAGCTCGTTTAAAC** |
| *spr28Δ* | AAAGAGCTACTATACGTACATAAAGTCAGTAAATAATCAA**CGGATCCCCGGGTTAATTAA** | ATTTCATATGTATCTAACGCTAACAAGGCCGTATATTTAT**GAATTCGAGCTCGTTTAAAC** |
| *VPH1-mCherry* | GGAAGTCGCTGTTGCTAGTGCAAGCTCTTCCGCTTCAAGC**CGTACGCTGCAGGTCGAC** | AGTACTTAAATGTTTCGCTTTTTTTAAAAGTCCTCAAAAT**ATCGATGAATTCGAGCTCG** |
| *NUP49-GFP*  *NUP49-mCherry* | GAATCGCCGTGTTACATCAAAAAACGAAAACACTGGCATCATTGAGCATA**cggatccccgggttaattaa** | AGACATTTGTACTTGTTATACGCACTATATAAACTTTCAGGGCGATTTAC**gaattcgagctcgtttaaac** |
