# Supplementary material for: Meiotic cellular rejuvenation is coupled to nuclear remodeling in budding yeast
Source: eLife. 2019 Aug 9;8:e47156. doi: 10.7554/eLife.47156 (PMC6711709; doi:10.7554/eLife.47156)
Supplement: Supplementary file 3. [file elife-47156-supp3.docx]

**Table S3. Plasmids used for strain construction.**

| **Plasmid Name** | **Description** |
| --- | --- |
| pUB4 | pFA6a-GFP(S65T)-KanMX6 |
| pUB72 | pFA6a-mCherry-KanMX6 |
| pUB73 | pFA6a-mCherry-NatMX6 |
| pUB76 | pFA6a-link-yeGFP-Kan |
| pUB217 | pFA6a-HphNT1 |
| pUB691 | pNH603-HIS3-VPH1-eGFP |
| pUB985 | pFA6a-3xeGFP-KanMX6 |
| pUB1104 | pLC605-pATG8-link-mKate-SPO20(51-91) |
| pUB1196 | pLC605-pARO10-eGFP-h2NLS-L-TM |
| pUB1197 | pYM28-mCherry-His3MX6 |
