# Supplementary material for: Meiotic cellular rejuvenation is coupled to nuclear remodeling in budding yeast
Source: eLife. 2019 Aug 9;8:e47156. doi: 10.7554/eLife.47156 (PMC6711709; doi:10.7554/eLife.47156)
Supplement: Supplementary file 4. — Transmission, exposure time, and excitation/emission wavelengths are specified for each channel. Distance between z-sections and number of z-sections acquired are indicated. [file elife-47156-supp4.docx]

| **Figure** | **RFP** | **GFP** | **BFP/DAPI** | **POL** | **Z Sectioning** |
| --- | --- | --- | --- | --- | --- |
| 3B-3G, 3 – S1A-B, 3 – S2, 3 – S3, 3 – S4, 3 – S5, 3 – S6A-B, 6 – S1A, 8A, 8 – S1A-B, 8 – S2A-B | 10%T, 0.025s EX: 575/25  EM: 632/60 | 10%T, 0.025s  EX: 475/28  EM: 523/36 | N/A | 32%T, 0.1s | 1 μm, 8 sections |
| 1B-1C, 1 – S1-S2, 2A-2B, 2E, 3H, 4A-4C, 5 – S1A-B, 6C-6D*, 6 – S1B, 6 – S2A-S2B, 6 – S3, 7A, 7C, 8 – S1C, 8 – S2C, 9A-B, 9D-E, 9 – S1A-B, 9 – S2A-B | 32%T, 0.025s EX: 575/25  EM: 632/60 | 10%T, 0.025s EX: 475/28  EM: 523/36 | N/A | 32%T, 0.1s | 1 μm, 8 sections |
| 5A-B, 6A**, 6B, 8C-D | 100%T, 0.1s  EX: 575/25  EM: 632/60 | 100%T, 0.1s EX: 475/28  EM: 523/36 | 100%T, 0.01s EX: 390/18  EM: 435/48 | 32%T, 0.1s | 0.2 μm, 40 sections |
| 8F | 100%T, 0.1s  EX: 575/25  EM: 632/60 | 100%T, 0.1s  EX: 475/28  EM: 523/36 | 100%T, 0.01s EX: 390/18  EM: 435/48 | 32%T, 0.1s | 1 μm, 8 sections |
| 4 – S1-S2** | 100%T, 0.1s  EX: 575/25  EM: 632/60 | 100%T, 0.1s  EX: 475/28  EM: 523/36 | 100%T, 0.1s EX: 390/18  EM: 435/48 | 32%T, 0.1s | 0.2 μm, 50 sections |

**Table S4. Imaging conditions.** Transmission, exposure time, and excitation/emission wavelengths are specified for each channel. Distance between z-sections and number of z-sections acquired are indicated.

*In 6C-6D, POL images were not acquired.

**In 6A, the early and late meiosis II cells were acquired with 0.2 μm over 30 sections.

***In 4 – S1 and 4 – S2, pre-meiosis cells were acquired with 0.3 μm over 33 sections.
