# Supplementary material for: Meiotic cellular rejuvenation is coupled to nuclear remodeling in budding yeast
Source: eLife. 2019 Aug 9;8:e47156. doi: 10.7554/eLife.47156 (PMC6711709; doi:10.7554/eLife.47156)
Supplement: Supplementary file 5. — Movies of strains with the indicated deletion, and either (1) a fluorescently tagged inner ring complex nucleoporin (Nup170-GFP) and a meiotic staging marker (Htb1-mCherry) or (2) a fluorescently tagged chaperone that marks age-induced protein aggregates (Hsp104-mCherry) and a gamete plasma membrane marker (yeGFP-Spo2051-91) were generated. For mutants with successful spore packaging, at least 25 tetrads were observed. For mutants with poor or unsuccessful spore packaging, at least 50 cells that proceeded through MII were observed and compared to wild type (UB11513 for Nup170-GFP; UB11821 for Hsp104-mCherry). [file elife-47156-supp5.docx]

| **Function** | **­Gene** | **NPC Strain**  **(Nup170-GFP)** | **Protein Aggregate strain (Hsp104-mCherry)** |
| --- | --- | --- | --- |
| Leading edge | *ady3Δ* | UB12414 | UB19758 |
|  | *don1Δ* | UB12461 | UB19756 |
|  | *irc10Δ* | UB12463 | UB19762 |
| Meiotic septins | *spr3Δ* | UB15307 | UB19752 |
|  | *spr28Δ* | UB15426 | UB19754 |
|  | *spr3Δspr28Δ* | UB15428 | UB19760 |

**Table S5. Meiotic septin and leading edge complex genes are not required for nuclear pore complex or protein aggregate sequestration.** Movies of strains with the indicated deletion, and either (1) a fluorescently tagged inner ring complex nucleoporin (Nup170-GFP) and a meiotic staging marker (Htb1-mCherry) or (2) a fluorescently tagged chaperone that marks age-induced protein aggregates (Hsp104-mCherry) and a gamete plasma membrane marker (yeGFP-Spo20^51-91^) were generated. For mutants with successful spore packaging, at least 25 tetrads were observed. For mutants with poor or unsuccessful spore packaging, at least 50 cells that proceeded through MII were observed and compared to wild type (UB11513 for Nup170-GFP; UB11821 for Hsp104-mCherry).
